# Current Treatment of Cleft Patients in Europe from a Provider Perspective: A Cross-Sectional Survey

**DOI:** 10.3390/ijerph191710638

**Published:** 2022-08-26

**Authors:** Inês Francisco, Gregory S. Antonarakis, Francisco Caramelo, Anabela Baptista Paula, Carlos Miguel Marto, Eunice Carrilho, Maria Helena Fernandes, Francisco Vale

**Affiliations:** 1 Institute of Orthodontics, Faculty of Medicine, University of Coimbra, 3000-075 Coimbra, Portugal; 2 Coimbra Institute for Clinical and Biomedical Research (iCBR), Area of Environment Genetics and Oncobiology (CIMAGO), Faculty of Medicine, University of Coimbra, 3000-075 Coimbra, Portugal; 3 Division of Orthodontics, University Clinics of Dental Medicine, University of Geneva, 1205 Geneva, Switzerland; 4 Laboratory of Biostatistics and Medical Informatics (LBIM), Faculty of Medicine, University of Coimbra, 3004-531 Coimbra, Portugal; 5 Clinical Academic Center of Coimbra (CACC), 3030-370 Coimbra, Portugal; 6 Centre for Innovative Biomedicine and Biotechnology (CIBB), University of Coimbra, 3000-075 Coimbra, Portugal; 7 Institute of Integrated Clinical Practice, Faculty of Medicine, University of Coimbra, 3004-531 Coimbra, Portugal; 8 Institute of Experimental Pathology, Faculty of Medicine, University of Coimbra, 3004-531 Coimbra, Portugal; 9 Faculty of Dental Medicine, University of Porto, 4200-393 Porto, Portugal; 10 LAQV/REQUIMTE, University of Porto, 4160-007 Porto, Portugal

**Keywords:** health disparities, cleft palate, cleft lip

## Abstract

The latest Eurocleft study reported several discrepancies in cleft care. Since then, no critical assessment has been performed. This study aimed to better understand the main strengths and inefficiencies of cleft care within Europe. The Google documents platform was used to create an online survey to investigate several aspects, i.e., provider characteristics, patient profile, services offered, and treatment protocols and complications. Descriptive statistics were calculated. The association between categorical variables was performed using Fisher’s exact test. The significance level chosen was 0.05. A total of 69 individuals from 23 European countries completed the survey. Centralized care was the preferred system, and the majority of the countries have an association for cleft patients and professionals (53.6%). The largest percentage of patients was seen in the university hospital environment (Fisher’s exact test *p* < 0.001). The majority of responders (98.6%) reported that an orthodontist was involved in cleft treatment, and 56.5% of them spend 76–100% of their time treating these patients. Despite cleft care having been reconfigured in Europe, a better consensus among the various centers regarding provider characteristics, services offered, and treatment protocols is still required. There is a need for better coordination between clinicians and national/international regulatory bodies.

## 1. Introduction

According to the World Health Organization, cleft care is still far from ideal due to service organization, inequalities of care, uncertainty around treatment, and limited resources [1]. Over the past decades, several improvements in cleft lip and/or palate (CLP) treatment have been made with some high-income countries adopting national protocols and the centralization of services, which have some advantages: standardized data collection; a coordinated approach; better responsiveness to patient and family needs due to interdisciplinary teams; and continuous monitoring [2,3,4,5].

In Europe, CLP affected 14.5 per 10,000 births between 2011 and 2018 according to the European Network for Epidemiological Surveillance of Congenital Anomalies report [6]. Cleft teams and treatment protocols for the management of patients with clefts vary considerably within and between European countries. In the late 1990s, Europe produced a set of recommendations for cleft care. This highlights that standardized procedures can allow for intercenter comparisons as well as the evaluation of treatment outcome in each individual center so as to aim for an improved in outcome should this be deemed necessary [1].

In 2001, Shaw et al. reported that some countries in Europe did not yet have team care, and they also found that 194 out of 201 teams had different surgical protocols for only unilateral clefts [2]. Later in 2011, a United Nations International Children’s Emergency Fund (UNICEF) survey in Bulgaria found that more than 40 percent of parents of children with clefts were advised to leave their children in an orphanage [7]. The complexity of cleft treatment, which includes multiple surgeries and medical treatments, may explain this finding. It is estimated that the average cost for cleft children up to the age of 10 is eight times higher than for healthy children of the same age [8]. Access to healthcare varies widely between and within countries, which can influence the provision of care in this respect. Cleft research has several challenges such as the length of follow-up and the range of different outcomes, since clefts can lead to disturbances in many neighboring structures and their associated functions [1]. Therefore, international collaboration is required to obtain a sample with adequate power [9,10,11].

The 2030 Agenda for Sustainable Development was launched in 2015 and defined 17 goals to be adopted by all United Nations Member States, recognizing the need to share knowledge, new technological advances, and financial resources to achieve the sustainable development goals in all countries. Goal 3 is focused on ensuring healthy lives and promoting well-being for all at all ages, and reads: “Achieve universal health coverage, including financial risk protection, access to quality essential health-care services and access to safe, effective, quality and affordable essential medicines and vaccines for all” [12]. In order to develop new sustainable strategies, fulfilling the aim of reducing inequalities in the 2030 Agenda, it is essential to recognize the current inefficiencies of cleft care. Despite the creation of the EUROCRAN project, which proposes to help international collaboration in Europe, the lack of evidence-based care in the field of orofacial clefting with only few randomized clinical trials being carried out is still a problem [13]. Furthermore, no recent efforts have been implemented to assess the current treatment approaches to CLP since the Eurocleft study in 2001. Therefore, this study aimed to instigate a critical appraisal of cleft care in Europe, which will allow for a better understanding of its main strengths and inefficiencies.

## 2. Materials and Methods

The study used a survey to investigate several areas of cleft care, namely, provider characteristics; patient profile; services offered; treatment protocols, especially orthodontic treatment; and complications related to orthodontic treatment. The development of this questionnaire began with a literature search to ensure content quality with the following keywords “cleft palate”, “treatment protocols”, “survey”, “orthognathic surgery”, and “orthodontics”. Subsequently, six experts, including a plastic surgeon, a maxillofacial surgeon, an orthodontist, a biostatistician, a researcher in the area of bone metabolism and regeneration, and an evidence-based medicine expert, were involved in the development process. Each question was constructed with the intention of avoiding unclear content (e.g., ambiguous words or double-barreled questions), guaranteeing the appreciation in the same way by all respondents. The Google documents platform was used to create an online survey with a unique URL, giving access to the survey at any time and anywhere in the world. The survey was available online from January 2021 until July 2021. For each question, participants placed a tick on their answer from a list provided. Appendix A presents the survey questions and answers.

A preliminary register was compiled through association membership lists with a known email address, namely Orphanet, European Cleft Organisations, the European Orthodontic Society, and the European Federation of Orthodontic Specialist Associations. Several medical specialties that are generally involved in cleft treatment were included, namely, maxillofacial surgeons, plastic surgeons, pediatric, orthodontists, speech and language. Subsequently, an email was sent to the mailing list established. Reminder emails were sent at 2, 4, and 8 weeks to nonresponders. After that, the survey was closed to new responses. The data was automatically stored using a “cloud” database. The unique study ID ensured the confidentiality of all data. An automated method then generated the numerical values, allowing data to be imported into the Statistical Package for the Social Sciences, version 24.0 for Windows (SPSS Inc., Chicago, IL, USA). Duplicate entries were rejected. Incomplete questionnaires were excluded. Descriptive statistics (frequency and, when applicable, means and standard deviations) were calculated for each question. The association between categorical variables was performed using Fisher’s exact test. The significance level was set at *p* < 0.05.

## 3. Results

### 3.1. Survey Responses and Covered Countries

A total of 79 out of 214 individuals on the mailing list responded to the survey (37%), of which 10 were excluded due to an incomplete questionnaire. There were 16 participants who decided not to participate because they do not treat patients with CLP. Overall, 69 individuals completed the entirety of the survey (56.5% female and 43.5% male), with a response rate of 36.7% (69 out of 188). The responding participants included 52 (75.4%) orthodontists, 9 (13%) maxillofacial surgeons, 5 (7.2%) plastic surgeons, 2 (2.9%) pediatric surgeons, and 1 (1.4%) speech and language therapist.

**Figure 1:**
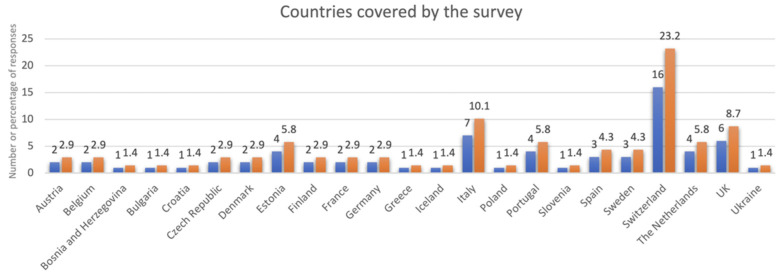
List of countries covered by the survey.

This survey includes a sample from 23 European countries of which Switzerland, the United Kingdom, and Italy had the highest response rate (Figure 1).

### 3.2. Providers’ Characteristics/Practice Management

The average number of years of professional experience was 21.6 (SD = 8.9). The respective centers/offices collaborated in the treatment of patients with CLP for an average of 31.1 years. An average of 73.9% of respondents work in a hospital and/or university environment.

Centralization of services (compared to local services) was the preferred reference system, with some countries (Bulgaria, Croatia, Czech Republic, Denmark, Finland, Slovenia, Sweden, and the Netherlands) reporting that it was the only reference system used (Table 1). Involvement of several medical specialties was reported, with orthodontics (98.6%) and maxillofacial surgery (81.2%) being the most frequently mentioned.

The majority of countries have a CLP association for patients and professionals (53.6%) (Table 1). However, five countries reported only parents’ associations (Czech Republic, Estonia, Finland, Iceland, and Slovenia). Of all 69 participants, 36 reported being a member of professional associations (52.2%).

### 3.3. Characterization of Patients

**Figure 2:**
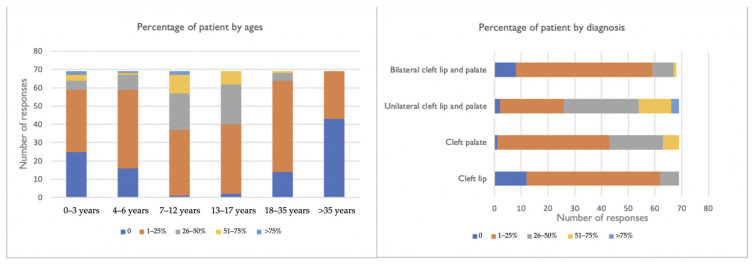
Distribution of patient by ages and diagnosis.

It was reported that the majority of patients lived less than 51 km away (46.4%) (Table 1). Most respondents reported that the majority of patients undergoing treatment were under 13 years of age (Figure 2). Hospital-based practices also had a statistically significant association with three age groups: 4–6 years (Fisher’s exact test, *p* = 0.01); 18–35 years (Fisher’s exact test, *p* = 0.04); over 35 years (Fisher’s exact test, *p* = 0.01). The remaining age groups do not differ in relation to the place of the delivery of services. The most prevalent diagnosis was unilateral cleft lip and palate (Figure 2), followed by isolated cleft palate, bilateral cleft lip and palate, and, finally, isolated cleft lip.

The largest percentage of patients were seen in a university hospital environment (Fisher’s exact test *p* < 0.001) (Table 2). An increase in the volume of patients was found, particularly in the university and/or hospital environment, when comparing current data with data from the last 3 years. Private practice did not register significant changes, evaluating 100 patients or less in most responses.

### 3.4. Treatment Protocols

**Figure 3:**
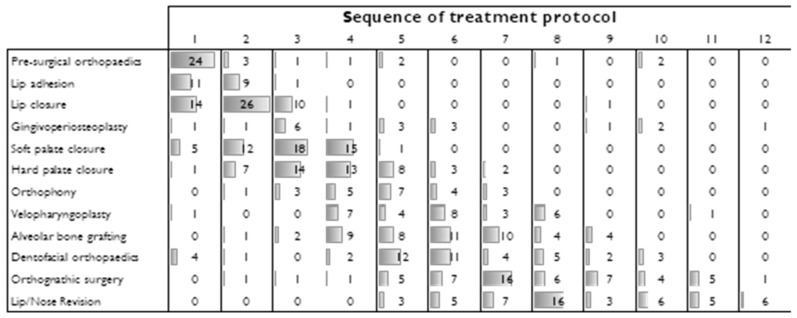
Sequence of treatment protocol. The numbers in the columns represent the responses given per procedure in chronological order.

Of the 69 responses, this survey showed 50 different treatment protocols. The most frequently reported protocol sequences were: pre-surgical orthopedics; lip closure; soft palate closure; hard palate closure; and dentofacial orthopedics. In most reported sequences, three procedures were consistently referred to: lip closure, soft palate closure, and hard palate closure (Figure 3). In contrast, the least reported procedure was gingivoperiosteoplasty (8 out of 50 possible sequences). On average, the centers performed 4.58 procedures in cleft treatment and only one center reported performing all the procedures listed.

Regarding orthodontic treatment, the majority of orthodontists spend approximately 76 to 100 percent of their time treating patients with CLP (Table 3). There is a statistically significant association (Fisher’s exact test, *p* < 0.001) between the time spent and the location of the office, with orthodontists in university hospital environments tending to spend more time on CLP treatment. The most used tool for orthodontic diagnosis was cone beam computed tomography (38.9%), but no statistically significant association (Fisher’s exact test, *p* = 0.05) was found between diagnostic tools and medical specialties. Overall, 70.9% of responding participants performed pre-surgical orthopedics, and unilateral and bilateral CLP were the phenotypes where it was most performed. The presence of reported complications from orthodontic or surgical treatment was relatively low (0–25%), with postoperative fistula being the most frequent complication following surgical procedures (Table 3).

## 4. Discussion

This survey attempted to understand the current situation with regard to cleft care in Europe since the Eurocleft study in 2000. In particular, years of professional and center experience, reference system, specialties involved in cleft treatment, presence of national associations, treatment protocols, and some concerns about orthodontic treatment were analyzed in this study.

A sample of 23 European countries were included in this study. Comparing this sample with the Eurocleft study, it was possible to ascertain: (1) inclusion of two countries: Bosnia and Herzegovina and Croatia; (2) no responses were obtained from the following countries: Slovakia, Romania, Norway, Latvia, Ireland, and Hungary [2]. Despite these differences, the authors feel that these studies are similar enough to allow us to compare the main strengths and inefficiencies in cleft care found. Furthermore, the high average of professional/center experience found in this survey (21.6/31.1 years) permits a reflection on the current practice of professionals who treat patients with CLP.

Regarding the reference system, few improvements were found since the Eurocleft study. Some countries chose to adopt centralized services—Finland, Bulgaria, and Spain; or a combination of localized and centralized services—Greece, Italy, Ukraine, Portugal, France, and Germany. Ness et al. suggested the development of centralized multidisciplinary services in the treatment of cleft patients in all countries since small centers with low samples have great difficulty in proving the quality of their outcomes [14]. To date, few adequately powered randomized clinical trials exist, and centralized services with or without a state-funded health system would allow for easier and more accurate clinical research.

Patients with CLP spend many years undergoing corrective procedures which require a multidisciplinary team. The majority of responders reported the involvement of several medical specialties in cleft treatment and this holistic approach is in line with American Cleft Palate-Craniofacial Association (ACPA) minimal standards for the cleft palate team (orthodontist, surgeon, and speech-language pathologist) [15]. Although the three most reported specialties are similar to the Eurocleft study (otorhinolaryngology, maxillofacial surgery, and plastic surgery), other specialties have been referred to as being important in cleft treatment, namely, orthodontics, dentistry, and phoniatrics/speech therapy. Studies have shown that there seems to be an association between its severity and the severity of the cleft, referring that some dental anomalies can be clinical markers and define subphenotypes of orofacial cleft [16]. The most common dental anomalies in CLP patients are: hypodontia, supernumerary teeth, anomaly of shape and size of the teeth, enamel mineralization, and ectopic eruption. Additionally, the abnormal craniofacial development can lead to the development of malocclusion, such as crossbite (anterior or posterior), open bite, skeletal Class III, and crowding [17]. These features may also increase the rate of oral colonization, which is related to a higher risk of dental caries and gingivitis, but these results are still controversial in the literature [18,19]. The role of the pediatric dentist occurs from infancy to adulthood and may include prenatal counseling, preventive dental care, pre-surgical maxillary orthopedics to realign the maxillary segments, infant orthopedics (e.g., maxillary expansion and/or protraction), orthodontic treatment, restorative dentistry, and prosthetic treatment. Thus, dental services are crucial to supervise craniofacial growth and development, preserve healthy dentition and gums, and correct jaw and occlusion discrepancies to achieve normal speech, hearing, and occlusion with a normal facial appearance and psychological well-being [17,20].

Furthermore, this survey demonstrated an improvement in the creation of national associations for parents and professionals in some countries, for example in Portugal. Based on the results of this survey, countries such as Bosnia and Herzegovina, Greece, Poland, and Ukraine still have no organized association. The creation of associations in these countries may help to spread accurate information to patients, families/caregivers, and professionals, and to identify essential characteristics of quality for team composition.

In general, the majority of patients with CLP are followed up in a university hospital environment. This contrasts with the 2018 study by Khavanin et al., who reported private practice (58.6%) as the current model of CLP orthodontic care provision in the United States of America (USA). The authors also reported that, in the responders’ opinion, the ideal model for orthodontic care is a combination of university/hospital and private practice [21]. In Europe, CLP treatment is often supported by National Health Service while in the USA only 2% of community orthodontists accept Medicaid, which can explain the differences in the model of care. According to Khavanin et al.’s study, financial and insurance concerns create difficulties in the delivery of cleft orthodontic care in the USA [21].

Based on the present results, hospital-based practices follow more patients in three specific age groups (4–6 years; 18–35 years; over 35 years). This may be explained because these age groups are usually associated with surgical procedures, namely, soft and hard palate closure at 4–6 years, orthognathic surgery at 18–35 years, and lip/nose revision in patients over 35 years.

Since the previous Eurocleft intercenter study, standardization of the cleft care protocol had been suggested as desirable. In the present survey, it found that heterogeneity in treatment protocols is still present (50 protocols for 69 responders), with the percentage in this study being 75% compared to 96% in 2000 [1]. These results were not expected, considering the 21-year interval between the two studies. Since timing and treatment sequences vary, the aggregation of data is complex, which explains the lack of supporting evidence in this field.

The majority of respondents (98.6%) reported that an orthodontist was involved in CLP treatment, with 56.5% of them spending 76–100% of their time treating patients with CLP. The ACPA study reported distinct results with only 32.8% of their orthodontists dedicating a majority of their time to cleft care [21]. These contradictory data may be associated with the place of service delivery, since in Europe orthodontists are integrated into a university hospital environment, while in the USA the majority of orthodontists are associated with private practice. Additionally, this may also explain why Europe seems to offer pre-surgical infant orthopedics more often than in the USA (70.9% vs. 48.6%) since orthodontists who are less experienced in managing patients with CLP are less likely to provide these treatments [21].

The most preferred diagnostic tool prior to orthodontic treatment was cone beam computed tomography (CBCT). Despite the Eurocleft study using lateral cephalograms, CBCT has become a diagnostic imaging tool in patients with CLP since it had a higher definition than conventional two-dimensional methods and lower radiation exposure compared with computed tomography [22,23]. The American Academy of Oral and Maxillofacial Radiology defined clinical recommendations regarding the use of CBCT and this included imaging in the context of CLP care, since these patients have medical conditions that required proper 3D analysis for accurate diagnosis [24].

As far as complications during orthodontic and surgical treatment are concerned, this study shows a low reported incidence of these complications, which is in accordance with a recent systematic review that showed an overall perioperative complication rate of 12.6%, with postoperative fistula being the most frequent complications [25]. Moreover, a retrospective study performed with 1408 patients concluded that the overall incidence of postoperative complications was 16.9% with a fistula rate of 13.6% [26]. Alvear et al. evaluated the complications of nasoalveolar molding and verified that despite the benefits of NAM exceeding the complications, this appliance had some complications that occurred mainly in the soft tissues, namely, irritation, ulceration, gingival, facial, or nasal bleeding [27].

The main limitation of most surveys is the low response rate, which was also relatively low in the present study. Considering the number of centers included in the last Eurocleft study, this survey represents around 34.3% of practicing providers in Europe. However, this number may be over or underestimated as some countries have reorganized the reference system. The rate and distribution of responses does not allow to draw conclusions about the current practice in cleft palate treatment for each European country. Even though we hoped the web-based approach would motivate more responses, the lack of compliance can be partly explained by the size of the questionnaire and the presence of few specialized centers for cleft care in Europe. However, the rate of responders is similar to the last Eurocleft study (40%), which allows a comparison of the results of both studies and highlights the current practice in cleft care among European countries. Nevertheless, the results of this study should be taken with caution as they do not represent all practicing professionals in Europe. Management of CLP patients requires an interdisciplinary team of specialists to achieve normal speech, hearing, and occlusion with a normal facial appearance and psychological well-being [28]. The timing and sequencing of treatment should be a result of the coordinated decision of a multidisciplinary team focusing on a patient-centered care and family’s needs approach. Most of the respondents were orthodontists (75.4%), so the results of this study may be biased from the perspective of this medical specialty.

The small sample size prevented a thorough factorial analysis to establish the validity of the questionnaire as a measurement instrument and the alpha of Cronbach considering all questions is 0.413, showing low reliability. Therefore, and as stated before, the results should be interpreted within the limitations.

This survey provides updated information on the current organization of cleft care within Europe. Based on the results, the achievement of a consensus with regard to cleft care across Europe remains suboptimal and challenging. These results are in agreement with a recent systematic review that concluded a lack of integrated high-quality clinical practice guidelines that can be used as universal guidelines in cleft treatment [29]. Therefore, there is a need for better coordination between clinicians and national and international regulatory bodies and centers. Studies focusing on how the providers’ characteristics and practice management issues influence the outcome of cleft treatment are still required. Further studies should focus on analyzing the association between the cleft services and clinical outcomes, considering child and parental satisfaction data. Those additional data with the results of this study will allow reconfigured cleft care services.

## 5. Conclusions

Despite cleft care having been reconfigured in Europe, a better consensus among the various centers regarding provider characteristics, services offered, and treatment protocols is still required. There is a need for better coordination between clinicians and national/international regulatory bodies.

## Figures and Tables

**Table 1:** Survey results on practice management.

Question		Response
Please select where your center/office is integrated:		
Hospital environment;		17.4%
University environment;		24.6%
Private practice;		26.1%
University hospital environment;		31.9%
What is the preferred reference system for patients with CLP in your country?		
Centralized services;		43.5%
Local services;		14.5%
Both;		36.2%
Not sure/None of the previous answers.		5.8%
How far from your center/office are the majority of your patients with CLP?		
0–50 km;		46.4%
51–100 km;		30.4%
101–150 km;		13.0%
>150 km.		10.1%
Select the medical specialties involved in CLP treatment in your department/office?		
Otorhinolaryngology		65.2%
Maxillofacial surgery		81.2%
Plastic surgery		50.7%
Neurosurgery		11.6%
Orthodontics		98.6%
Pediatric surgery		30.4%
Phoniatrics/Speech Therapy		72.5%
Dentistry		71.0%
Geneticist		47.8%
Child and adolescent psychiatry		42.0%
Clinical nurse specialist		42.0%
Are there national CLP associations in your country?		
Yes, only for professionals;		13.0%
Yes, only for parents;		17.4%
Yes, for both;		53.6%
No or not sure.		15.9%

**Table 2:** Survey results on distribution of patients with CLP by office environment.

		**How Many Patients with CLP in the Last 3 Years**			
		**0–100**	**101–200**	**201–300**	**>300**
Office is in	Hospital	3	1	3	5
	University	11	3	2	1
	Private practice	17	0	1	0
	University hospital	7	4	1	10
		**How Many Patients with CLP Currently**			
		**0–100**	**101–200**	**201–300**	**>300**
Office is in	Hospital	5	0	0	7
	University	12	2	0	3
	Private practice	16	1	1	0
	University hospital	4	3	5	10

**Table 3:** Survey results on orthodontic care.

Question	Response
What percentage of the time is an orthodontist present at your multidisciplinary clinic?	
0–25%;	21.7%
26–50%;	13.0%
51–75%;	8.7%
76–100%.	56.5%
What is the most used tool for orthodontic diagnosis and treatment planning in your center/office for patients with CLP?	
Panoramic and cephalometric radiograph;	31.3%
Cone beam computed tomography;	38.9%
Computed tomography;	24.3%
Other	5.6%
How often do you perform pre-surgical orthopedics on patients with CLP in your clinical practice?	
Always;	18.8%
Often;	30.4%
Sometimes;	21.7%
Never.	29.0%
In which cleft phenotypes do you perform pre-surgical orthopedics?	
Cleft lip	17.4%
Cleft palate	29.0%
Unilateral cleft lip and palate	56.5%
Bilateral cleft lip and palate	62.3%
Pierre Robin sequence	36.2%
Other	13.0%
What percentage of patients with CLP have complications during orthodontic or surgical treatment?	
0–25%;	90%
26–50%;	9%
51–75%;	1%
76–100%.	0%
What is the most frequent complication of patients with CLP after surgical procedures:	
Postoperative fistula;	43%
Postoperative infection;	10%
Postoperative airway complication;	9%
Revision;	13%
Other	32%

## Data Availability

The data presented in this study are available on request from the corresponding author.

## Appendix A

**Table A1:** Survey questions and answers options.

Question	Answer Options (If Applicable)
In what country do you practice?	
What is your specialty (orthodontics, maxillofacial surgery, other)?	
How many years of professional experience do you have?	
How many years does your centre/office collaborate in CLP treatment?	
Please select where your centre/office is integrated:	Hospital environment;
	University environment;
	Private practice;
	University hospital environment;
What is the preferred reference system for patients with CLP in your country?	Centralization of services;
	Local services;
	Both;
	Not sure/None of the previous answers.
How far from your centre/office are the majority of your patients with CLP?	0–50 km;
	51–100 km;
	101–150 km;
	>150 km.
How many CLP patients have been treated at your centre/office over the past 3 years?	0–100; 101–200; 201–300; >300.
How many CLP patients are currently under treatment at your centre/office?	1–50; 51–100; 101–150; >150.
Please tick the approximate percentage of your patient population in each diagnosis:	Cleft lip Cleft palate Unilateral cleft lip and palate Bilateral cleft lip and palate
Select your centre/office’s CLP treatment protocol in chronological order.	Pre-surgical orthopaedics Lip adhesion Lip closure Gingivoperiosteoplasty Soft palate closure Hard palate closure Orthophony Velopharyngoplasty Alveolar bone grafting Dentofacial orthopaedics Orthognathic surgery Lip/Nose Revision
Select the medical specialties involved in CLP treatment at your department/office?	Otorhinolaryngology
	Maxillofacial surgery
	Plastic surgery
	Neurosurgery
	Orthodontics
	Paediatric surgery
	Phoniatrics/ Speech therapy
	Dentistry
	Geneticist
	Child and adolescent psychiatry
	Clinical nurse specialist
Are there national CLP associations in your country?	Yes, only for professionals;
	Yes, only for parents;
	Yes, for both;
	No or not sure.
Are you member of any cleft palate association?	Yes No
What percentage of the time is an orthodontist present at your multidisciplinary clinic?	0–25%;
	26–50%;
	51–75%;
	76–100%.
What percentage of your orthodontist’s practice is devoted to the care of patients with CLP	0–25%;
	26–50%;
	51–75%;
	76–100%.
What is the most used tool for orthodontic diagnosis and treatment planning in your centre/office for patients with CLP?	Panoramic and cephalometric radiograph;
	Cone beam computed tomography;
	Computed tomography;
	Other
How often do you perform pre-surgical orthopaedics in patients with CLP in your clinical practice?	Always;
	Often;
	Sometimes;
	Never.
In which cleft phenotypes do you perform pre-surgical orthopaedics?	Cleft lip
	Cleft palate
	unilateral cleft lip and palate
	bilateral cleft lip and palate
	Pierre Robin sequence
	Other
What percentage of patients with CLP have complications during orthodontic or surgical treatment?	0–25%;
	26–50%;
	51–75%;
	76–100%.
What is the most frequent complication of patients with CLP after surgical procedures:	Postoperative fistula;
	Postoperative infection;
	Postoperative airway complication;
	Revision;
	Other
